# How Do Collective Efficiency and Norms Influence the Social Resilience of Iranian Villagers Against the COVID-19? The Mediating Role of Social Leadership

**DOI:** 10.3389/fpubh.2022.861325

**Published:** 2022-04-01

**Authors:** Naser Valizadeh, Ezatollah Ghazani, Morteza Akbari, Javad Shekarkhah

**Affiliations:** ^1^Department of Agricultural Extension and Education, School of Agriculture, Shiraz University, Shiraz, Iran; ^2^Department of Agricultural Extension and Education, College of Agriculture, Tarbiat Modares University, Tehran, Iran; ^3^Faculty of Entrepreneurship, University of Tehran, Tehran, Iran; ^4^Department of Accounting, Faculty of Management and Accounting, Allameh Tabataba'i University, Tehran, Iran

**Keywords:** collective efficacy, norms, social resilience, social leadership, COVID-19

## Abstract

The main purpose of the present research was to investigate the effects of collective efficacy and norms on the social resilience against the COVID-19 with the mediating role of social leadership. To this end, a cross-sectional survey was carried out in the Kerman and Fars provinces of Iran. Finally, 206 villagers were selected as the sample for collecting the required information. The research tool was a close-ended questionnaire whose validity and reliability was evaluated and confirmed. The results of testing direct hypotheses using structural equation modeling revealed that collective efficacy, social leadership, and norms had significant positive effects on social resilience against the COVID-19 pandemic. Comparison of the standardized effects demonstrated that collective efficacy is the most powerful predictor of the social resilience of villagers. Furthermore, testing indirect (mediation) hypotheses revealed that social leadership can successfully mediate the effect of collective efficacy on social resilience against the COVID-19. Investigating the moderated indirect hypotheses showed that governmental supports moderated the effect of collective efficacy on social resilience. Taken together, the independent variables could account for 62% of social resilience variance change. In the end, the practitioners, decision-makers, and interveners of the COVID-19 management programs in rural communities were provided with some applicable recommendations to be able to foster social resilience against the COVID-19.

## Introduction

When the COVID-19 outbreak was introduced as a pandemic by the WHO in early 2020, there were widespread waves of concern and tension at the local, national, and international levels. This epidemic and its high rate of infection have had devastating and damaging impacts on the quality of life of human societies around the world (1). COVID-19 was first observed in Wuhan, China, and spread out rapidly in various countries of the globe. In addition to its impacts on the health and economic systems of global societies, COVID-19 has also posed serious psychological, physical, environmental, and cognitive dimensions (2, 3). The disease has also led to significant changes in health care, transportation, and education systems (4, 5). The COVID-19 pandemic has forced many social groups to change their lifestyles to reduce its negative impacts (6, 7). Villagers have also been one of the vulnerable groups to coronavirus shock who have tried to improve their resilience to the negative impacts of the disease by using different strategies (8). Such shocks have had direct and indirect effects on rural and agricultural areas (9). In some cases, the timing of the outbreak has been one of the most important barriers to buying and selling agricultural and non-agricultural products of villagers (10). Because of the transportation restrictions and COVID-19 lockdowns, the products produced by the villagers remain in the village or on their farms. These delays in the timely sale of products lead to corruption or economic losses (11).

During an epidemic, factors such as mortality, social distancing regulations, and mobility reduce access to labor in rural areas. Thus, it negatively affects an important part of the food security chain in rural areas (12). A review of the research literature (12–15) shows that rural communities' decisions about coping and resilience with different shocks are a complicated process. This process generally depends on various factors such as comprehensive psychological, economic, and social factors (5, 16–20). However, most of these studies include various shocks such as water scarcity (18), drought (19), floods (17), landslides (20), and climate change (16), but specifically in the field of COVID-19, few studies have focused on the role and importance of psychological and social factors. In other words, very few studies have been conducted on the resilience of rural communities to the COVID-19 pandemic, especially in developing countries. To bridge this gap, it was logical to research the resilience of rural communities against the COVID-19 pandemic and its social and psychological determinants.

Socio-psychological factors play a very important role in the resilience of rural communities against epidemics such as COVID-19 (12). It should be mentioned that the study of these socio-psychological factors in rural communities plays a key role in highlighting and managing issues related to the wider consequences of pandemics such as fear. Such consequences can be observed in various areas such as livelihood, employment, health services, and security (6). From the perspective of corona shock resilience, it should be emphasized that examining the socio-psychological factors of villagers can help to adopt new adaptation strategies or modify existing adaptation options (21). Socio-economic intervention policies and programs in rural areas are likely to fail during the COVID-19 epidemic if rural socio-economic resilience strategies and determinants are not addressed (6, 12), in this regard, identifying and examining the key socio-psychological factors that can be effective in directing policies and programs of resilience against the COVID-19 pandemic are of great importance in developing countries. As mentioned earlier, many studies have been conducted on socio-psychological factors predicting resilience and adaptation to various shocks (especially environmental shocks) [see (5, 17–19)]. However, to our best knowledge, no study examines the socio-psychological factors affecting the resilience of rural communities to the COVID-19 pandemic. Therefore, in this study, an attempt was made to fill this research gap by examining the socio-psychological factors affecting the resilience of rural communities against the COVID-19 pandemic in Iran. To achieve this aim, we used a conceptual framework, the development stages of which are discussed in section Theoretical Background and Development of Hypotheses. In balance, this study is original from several perspectives. First, to our best knowledge, no similar study has been conducted in Iran and around the world. Second, using a literature review, a socio-cognitive framework was designed to predict the social resilience of villagers in this study, which seems to play a key role in strengthening social resilience. Third, the variable of governmental support was considered as a moderator of the relationships of social leadership, collective efficiency, and norms with social resilience. Furthermore, social leadership mediated the relationships of collective efficiency and norms with social resilience. These mediating and moderating variables resulted in a model that could explain the mechanisms of social resilience formation more accurately.

## Theoretical Background and Development of Hypotheses

### Resilience Against the COVID-19 Pandemic

Resilience theory entered the research literature of many scientific disciplines from the ecology field (1, 16, 22, 23). In its evolution, this theory used the foundations of theories such as complexity theory, agent-based theory, and systems of systems theory (24). This theory is based on the basic assumption that different social, economic, and ecological systems face a series of uncertainties that make it difficult to predict patterns and trends (22, 25). In other words, in these systems, there is a variable set of shocks and serendipities such as floods, droughts, and diseases that are part of the facts (1, 26).

There are many definitions of the concept of resilience. Many of these definitions define resilience of a system as the capacity of that system to withstand and/or adapt to disturbances over time (25, 27, 28). This resistance and adaptation should be such that the system maintains its functions and has no problem in providing services to the stakeholders involved in that system (16, 23). Borrion et al. (25) argue that resilient systems have three salient features: (1) they have a high capacity for resistance to change and adversity, (2) they have a high ability in self-organization, and (3) they have a high ability to learn and adapt. The variety of definitions and the wide range of applications of this concept have led to the presentation of different dimensions and indicators to measure this phenomenon in various studies [see (1)]. In a study, Kumpfer (29) introduced five sub-indicators for measuring resilience: cognitive factors, spiritual factors, behavioral factors, physical factors, and emotional factors. Maleksaeidi et al. (16) claim that adaptability, diversity, learning, diversity, and self-organization opportunity are key dimensions for the resilience of farm-households against climate change. Some researchers [see (30–33)] consider social capital as one of the most important dimensions of societies' social resilience to shocks.

Beyond all the physical and structural aspects that can be considered in defining and explaining the concept of resilience, the social dimensions of resilience/social resilience have been less considered in studies (1, 34). Social resilience can be interpreted as the level of the human capacity to anticipate, resist, manage, adapt, and recover from crises (35). In the event of new and devastating shocks such as COVID-19, communities' spirit of participation and social cohesion can have a significant effect on reducing vulnerability and the harmful effects of epidemics. Accordingly, social resilience against the COVID-19 pandemic can help sustain the functional capacity of communities such as rural communities (1, 32, 33). According to Alizadeh and Sharifi (1) and Ghazani et al. (36), social cohesion, social trust, social participation, and social relationships were introduced in this study as four main dimensions of social resilience of rural communities against the COVID-19. Social cohesion refers to the degree of interaction, cooperation, conflict, and differences between local/rural people in the context of the COVID-19 pandemic. The degree to which local people trust relatives, locals, strangers, and governmental and non-governmental entities in the management of COVID-19 is called social trust. The degree of subjective and objective participation of local people in the process of managing the coronavirus epidemic is social participation. It should be noted that social relationships refer to the level of communication and cooperation between the various stakeholders of the disease management process. In this research, according to the main purpose, social resilience against the COVID-19 was considered as the main dependent variable. Therefore, the effect of predicting variables (collective efficiency, norms, social leadership, and government support) on it was measured and analyzed.

### Collective Efficacy

According to the social identity models of collective action, collective efficiency is one of the most important predictors of individuals' behaviors in the face of various crises and shocks (37–39). This concept is usually used in conjunction with the concept of self-efficacy (40). Self-efficacy is a person's belief in his or her ability to succeed in a particular situation (41, 42). Bandura (40) argues that the collective efficacy of individuals can affect members' goals of behavior, resource management, and social trust. In general, collective efficiency refers to individuals' perceptions of the effectiveness of collective actions or tasks to solve a particular problem (43, 44). In other words, the greater the perceived collective efficacy in dealing with COVID-19 among the community (villagers), the greater their social resilience to this shock. Although the effectiveness of collective efficacy in improving the resilience of different social groups has been emphasized by others [see (45–47)], primary searches show that no study has examined the effect of perceived collective efficiency on the resilience against the COVID-19 in rural communities. The study of Yazdanpanah et al. (12) is one of the few related studies in this field. However, in this study, they have examined the effect of collective efficacy on COVID-19 coping styles, not social resilience. In this regard, this variable was considered as one of the key variables explaining the social resilience of villagers. It should be noted that in the present study, the perceived collective efficiency in the field of COVID-19 in addition to the direct effect indirectly (through social leadership) affects the social resilience of the villagers. Based on the abovementioned debates, we hypothesize that:

H1: Collective efficacy has a significant influence on social resilience against the COVID-19.
H2: Collective efficacy has a significant influence on social leadership against the COVID-19.

### Norms (Social and Moral)

Norms are thought models or guidelines by which we control and evaluate the actions of ourselves and others. Internal norms are norms that if not observed, there is no formal and specific punishment. External/social norms are norms that are predetermined for members of society. Fear of punishment and inner desire motivate members of society to follow the norm (48). If norms are not stable in society or are in conflict with some other social orders, people in the society will follow the norms less (49). A review of the research literature shows that different types of subjective (social) and moral norms have been used to analyze the behavior of individuals in the face of various shocks [see (18, 50)]. According to researchers (51–53), subjective (social) norms are one of the drivers of preventive behaviors for COVID-19. Subjective norms of COVID-19 refer to the level of external pressure perceived by villagers to take specific actions such as the use of preventive measures (53). In other words, subjective norms refer to the perceived evaluation of a person's behavior by the community and/or those around him/her. The greater the perceived behavioral control, the greater a person's resilience against the COVID-19 pandemic. In addition to subjective norms, researchers [see (50, 54, 55)] also emphasize the moral considerations as a significant driver of preventive behavior and resilience against the COVID-19 pandemic. In such circumstances, individuals may view health protocols and participation in COVID-19 management as a personal commitment or moral responsibility for themselves (55). It can be concluded that normative considerations may play a vital role in explaining and predicting preventive behaviors and adopting resilience strategies against the COVID-19 pandemic. Therefore, this variable is considered as one of the factors affecting the resilience of villagers against the COVID-19 pandemic. It is worth mentioning that given that norms may theoretically lead to the strengthening or weakening of shared/social leadership, the variable of shared leadership was considered as a mediator between norms and resilience against the COVID-19 pandemic. Thus, we hypothesize that:

H3: Norms have a significant influence on social resilience against the COVID-19.
H4: Norms have a significant influence on social leadership against the COVID-19.

### Social Leadership

Researchers have come up with very different definitions of the concept of shared leadership. In this study, the concept of social leadership and shared leadership are considered synonymous. This concept was first developed in 1954 by Gibb. One of the most well-known definitions of shared leadership has been provided by Pearce and Conger (56). These researchers consider shared leadership as the collective or mutual influence of members of society on each other (57, 58). Shared leadership is a process of interactive influence between members of communities. The purpose of this type of leadership is to help each other achieve the collective goals of the community or group (58). In a meta-analytic study, Mukundi Gichuhi (59) examined the relationship between shared leadership and organizational resilience. The results of this study showed that shared leadership is one of the important variables that can positively affect resilience in different organizations and communities. This result has been supported by other researchers [see (1, 60)]. Specifically, in the case of the COVID-19 pandemic, it can be said that the shared/social leadership reflects the degree of influence and cooperation of the members of the rural community with each other in the field of the COVID-19 pandemic. By strengthening shared leadership in rural society, the resilience to disease shock increases (1). Thus, shared/social leadership in the context of COVID-19 was introduced as one of the main potential predictors of the social resilience of the villagers against the shock of COVID-19. Thus, we hypothesize that:

H5: Social leadership has a significant influence on social resilience against the COVID-19.
H6a: Social leadership mediates the relationships among collective efficacy and social resilience against the COVID-19.
H6b: Social leadership mediates the relationships among norms and social resilience against the COVID-19.

### Governmental Supports

According to the International Fund for Agricultural Development (3), governmental support is always an important part of post-shock agricultural and rural development interventions programs that can significantly contribute to the resilience of social systems. Lee and Lemyre (61) and Ratnasingam et al. (62) state that government support can affect the resilience responses and behaviors of individuals in the face of the risks and shocks. In other words, government support allows individuals to have minimal required options for economic responses, at least in the early stages of shocks such as COVID-19 (63, 64). The shock of COVID-19 also left a lot of social, economic, and environmental damage in rural communities of different countries, especially developing and underdeveloped countries (12). Rural and agricultural communities in these countries generally have low incomes, therefore, they are widely grappling with the negative consequences of this crisis (3). Government support, however, can increase their resilience to the COVID-19 shock and make them less vulnerable to the effects of the epidemic (64). In this regard, government support was also considered as one of the factors that can affect social resilience. However, in this study, government support was considered as a moderator of the relationship between collective efficiency, norms, and shared leadership with social resilience. Thus, we hypothesize that:

H7a: Governmental support moderates the link between collective efficacy and social resilience against the COVID-19.
H7b: Governmental support moderates the link between social leadership and social resilience against the COVID-19.
H7c: Governmental support moderates the link between norms and social resilience against the COVID-19.

**Figure 1:**
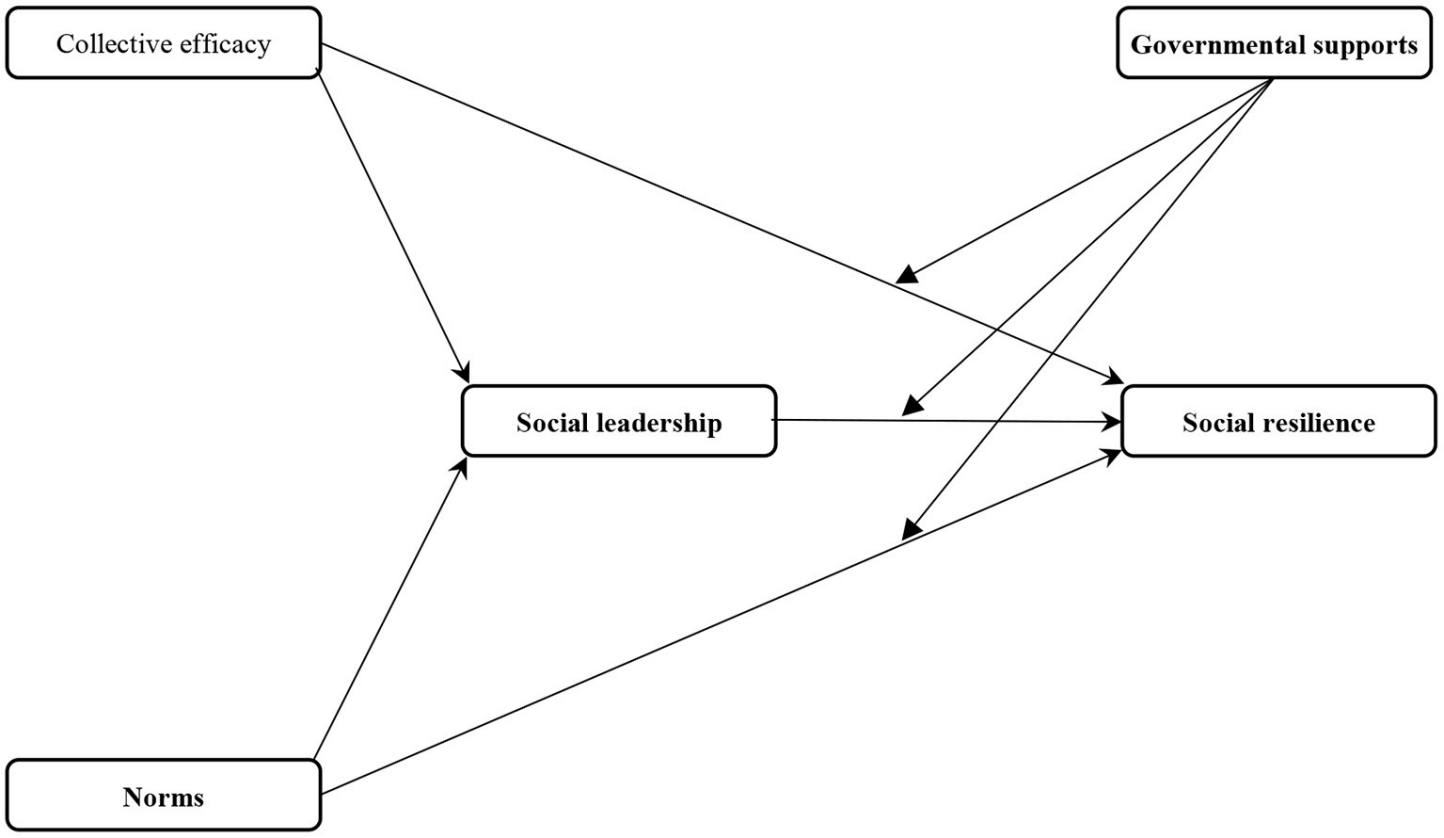
Research model.

Figure 1 demonstrates the proposed research framework of social resilience against the COVID-19 in rural communities.

## Methodology

### Research Typology

This research is an applied and quantitative study. Therefore, its results can be used by various end-users such as villagers, academic researchers, planners, and decision-makers at different levels of the COVID-19 pandemic management programs. In other words, it contributes to the development of resilience programs for rural communities in developing countries such as Iran.

### Study Area, Population, and Sampling Method

The population of this study was the villagers of Sirjan and Eghlid counties in the Kerman and Fars provinces of Iran. These two provinces are located in the south of Iran. There were three main reasons for choosing cases from this area. First, increasing the social resilience against COVID-19 disease was one of the main research priorities of the Government of the Islamic Republic of Iran. In other words, since the onset of the disease, the Iranian government has encouraged researchers to examine the social resilience of villagers and the factors affecting it. Second, according to the Ministry of Agricultural Jihad, the level of social resilience against Corona was low among villagers in Fars and Kerman provinces. In this regard, conducting research that can identify some of the socio-psychological variables affecting it was of great importance. Third, due to the mobility constraints imposed at the time of this study, the authors were only able to collect data from these two provinces and did not have access to the other provinces. According to the 2016 census, 101,934 people live in the villages of these two counties (35,159 villagers in Eghlid and 66,775 villagers in Sirjan). Cochran's formula was used to estimate the required sample size. Cochran's formula estimated the required sample size at 206 people. The samples were selected using a multi-stage sampling method. In the first stage, the villagers of Sirjan and Eghlid were purposefully selected as the study population. The most important reason for choosing these two counties as the study population was the ease of access of researchers to the study community. In the second stage, to select a representative sample from each of these two counties, several sub-counties were randomly selected. In the third stage, one village was randomly selected from each of these sub-counties. In other words, the respondents were selected from among the villagers based on the sub-counties.

### Measurement of Constructs

The four constructs [social resilience against the COVID-19 pandemic (SRCS), social leadership in epidemic conditions (SLEC), collective efficiency in disease control (CEDC), and norms about the COVID-19 (NC)] used in Figure 1 were measured through a five-point Likert scale (1: strongly disagree to 5: strongly agree). A five-level Likert scale was also used to measure governmental supports (GS) as the fifth construct used in the theoretical framework. However, the labels used to measure its items were 1: very low to 5: very high. To measure SRCS, SLEC, CEDC, NC, and GS 14, 3, 3, 3, and 5 items were applied, respectively (Table 1). The items of SRCS were adapted from Ghazani et al. (36). The items of CEDC, NC, and GS with some changes and corrections were taken from the study of Yazdanpanah et al. (12), and Savari and Gharechaee (66). It should be mentioned that all the items used to measure the variables NC and SLEC were adapted from Salas Vallina et al. (58) and Chiu et al. (65).

**Table 1:** Items measuring SRCS, SLEC, CEDC, NC, and GS and corresponding alpha coefficients.

**No**.	**Items**	**Sources**
**SRCS (Social participation): (α** **=** **0. 73)**		(36)
1	Since the beginning of the Corona epidemic, I have tried to participate in social activities to solve my village's problems.	
2	Since the beginning of the Corona epidemic, I have tried to help the activities of relevant institutions such as health centers, rural administration centers, and the Islamic Council voluntarily.	
3	Since the beginning of the Corona epidemic, I have been actively involved in implementing health and disease-related initiatives.	
4	I welcome the presence of government agencies and their agents in the village to facilitate the fight against Corona.	
**SRCS (Social trust): (α** **=** **0.77)**		(36)
1	I have always been encouraged by the help of other villagers to get out of the Corona crisis.	
2	I have always been encouraged by my family members to get out of the Corona crisis.	
3	The presence of village elders alongside the people during the Corona period has created trust and empathy among the people.	
**SRCS (Social cohesion): (α** **=** **0.81)**		(36)
1	During the Corona epidemic, the villagers do not hesitate to help each other.	
2	All villagers are united in eradicating the disease and breaking the transmission chain.	
3	To deal with the negative effects of the coronavirus, I consult with friends and other villagers.	
4	The problems of the medical staff in the village during the Corona epidemic are like our own problems and I try to help them as much as I can to solve these problems.	
**SRCS (Social relationships): (α** **=** **0.82)**		
1	Village government agencies are pursuing programs to cope with the COVID-19 pandemic.	(36)
2	People had good contact with government and local institutions and their representatives during the Corona epidemic.	
3	Village public institutions are actively involved in raising awareness and quality of health services.	
**Social leadership in epidemic conditions (SLEC): (α** **=** **0.73)**		(58, 65)
1	I try to spend time guiding those around me and the villagers about the COVID-19 pandemic.	
2	I teach new things I know about the COVID-19 disease.	
3	I take the lead to increase the participation of others in collective activities to deal with the crisis.	
**Collective efficiency in disease control (CEDC): (α** **=** **0.71)**		(12)
1	I believe that we need mutual help from other members of society to eradicate the epidemic.	
2	Reducing the side effects of the COVID-19 pandemic is easy for me.	
3	I believe I can control the effects of the COVID-19 pandemic in the village.	
**Norms about COVID-19 (NC): (α** **=** **0.82)**		(66)
1	If I follow the COVID-19 health protocols, I will be approved by those around me.	
2	Participating in epidemic management is a moral duty for each of us villagers.	
3	Active participation in the COVID-19 management practices is commonplace among villagers.	
**Governmental supports (GS): (α** **=** **0.74)**		(12)
1	Receiving assistance from the government to provide agricultural/livestock inputs.	
2	Extension of the loan repayment period.	
3	The strict control of entry/exit from villages.	
4	Severe quarantine and closure of high-risk jobs.	
5	Being provided with governmental subsidies for livelihood assistance.	

### Validity and Reliability of Research Tool

The tool used to collect the required information on the social resilience of villagers and the factors affecting it was a researcher-made and close-ended questionnaire. The face and content validity of the questionnaire was evaluated and confirmed using the opinions of an expert group. These experts raised some points on the questionnaire and we tried to address them point by point. Then, a pilot study was conducted with 30 villagers. After the pilot test, Cronbach's alpha coefficients were used to evaluate the reliability. Table 1 shows the reliability of the items measuring the variables used in the framework. At this stage, some corrections were made to the questionnaire. After removing the ambiguities and shortcomings of the questionnaire based on the results of the pilot study, the questionnaire was applied for the main cross-sectional survey of villagers in Sirjan and Eghlid counties. After conducting a cross-sectional survey to collect the required data, the composite reliability indices, loading factors of items (in the first-order confirmatory factor analysis), and average variance extracted (AVE) were employed as the main reliability and validity analysis criteria.

### Data Collection and Analysis

Data collection was done by the first and second authors. In data collection, they used two groups of data collection. The first group was employed to collect information from the villagers of Eghlid County and the second group was employed to collect information from the villagers of Sirjan County. Each of these groups consisted of four members with experience in collecting cross-sectional information. Data collection was performed from November 15 to December 15, 2021. According to the estimated sample size, 206 villagers were interviewed in different villages. Eight questionnaires were discarded due to deficiencies in the answers. Finally, 198 questionnaires were analyzed. Data analysis was performed using Smart PLS_3_ software.

## Results

### Correlation Between Variables

Table 2 summarizes the correlations among the variables used in the theoretical framework. The results of correlation analysis implied that the variables social leadership (r = 0.689; *p* < 0.01), collective efficacy (r = 0.671; *p* < 0.01), norms (r = 0.625; *p* < 0.01), and governmental supports (r = 0.601; *p* < 0.01) were positively and significantly correlated with SRCS. Comparison of correlations between variables shows that collective efficacy and social leadership have the highest correlation values with social resilience against the COVID-19 pandemic, respectively. In addition, collective efficacy (r = 0.519; *p* < 0.01) and norms (r = 0.456; *p* < 0.01) also had significant positive correlations with social leadership. Nevertheless, the correlation value obtained for collective efficacy was higher than the corresponding value for norms.

**Table 2:** Correlations among the study variables.

	**SRCS**	**SLEC**	**CEDC**	**NC**	**GS**
SRCS	1				
SLEC	0.689^**^	1			
CEDC	0.671^**^	0.519^**^	1		
NC	0.625^**^	0.456^**^	0.524^**^	1	
GS	0.601^**^	0.362^**^	0.656^**^	0.485^**^	1

*SRCS, Social resilience against the COVID-19 pandemic; SLEC, Social leadership in epidemic conditions; CEDC, Collective efficiency in disease control; NC, Norms about COVID-19; GS, Governmental supports*.

** *Sig. level: 0.01 error*.

### Measurement Models of the Constructs

Table 3 represents the results of measurement models of the constructs. Based on the results of this section of structural equation modeling, the loading factors for all items used to measure social leadership, collective efficacy, norms, governmental supports, and social resilience against the COVID-19 pandemic were above the acceptable value of 0.4. According to Hair et al. (67), acceptable loading factors in the measurement models are usually >0.4. The values obtained for CR and AVE indices for all variables were higher than 0.7 and 0.5, respectively. This result means that CR and convergent validity have been at the appropriate level. In addition, the rho-A criterion was also employed to evaluate the reliability of the construct. According to Azar et al. (68), the acceptable cut-off value for this criterion is 0.7. Because all rho-A values were >0.7 in the present study, the reliability of constructs and items measuring them were proved. Fornell-Larcker Criterion was applied to evaluate the discriminant validity of the construct. Statistically, if the AVE for each variable is greater than the highest squared correlation value of that variable with the other variables, it can be argued that discriminant validity has been confirmed (68). The results of this study showed that the AVE values of all structures are higher than the greatest squared correlations. Therefore, discriminant validity was confirmed. Overall, the results of Table 3 show that the collected data can be used for structural analysis.

**Table 3:** Evaluation of measurement models and the reliability, validity, and normality of assessment.

**Items/variables**	**SRCS**	**SLEC**	**CEDC**	**NC**	**GS**
SRCS1	0.947				
SRCS2	0.415				
SRCS3	0.938				
SRCS4	0.925				
SLEC1		0.685			
SLEC2		0.764			
SLEC3		0.621			
CEDC1			0.682		
CEDC2			0.757		
CEDC3			0.717		
NC1				0.678	
NC2				0.767	
NC3				0.645	
GS1					0.695
GS2					0.503
GS3					0.615
GS4					0.585
GS5					0.700
CR	0.89	0.73	0.76	0.74	0.76
rho-A	0.92	0.76	0.73	0.75	0.86
AVE	0.70	0.50	0.51	0.50	0.51

*SRCS, Social resilience against the COVID-19 pandemic; SLEC, Social leadership in epidemic conditions; CEDC, Collective efficiency in disease control; NC, Norms about the COVID-19; GS, Governmental supports*.

### Testing Hypotheses Using a Structural Model

**Figure 2:**
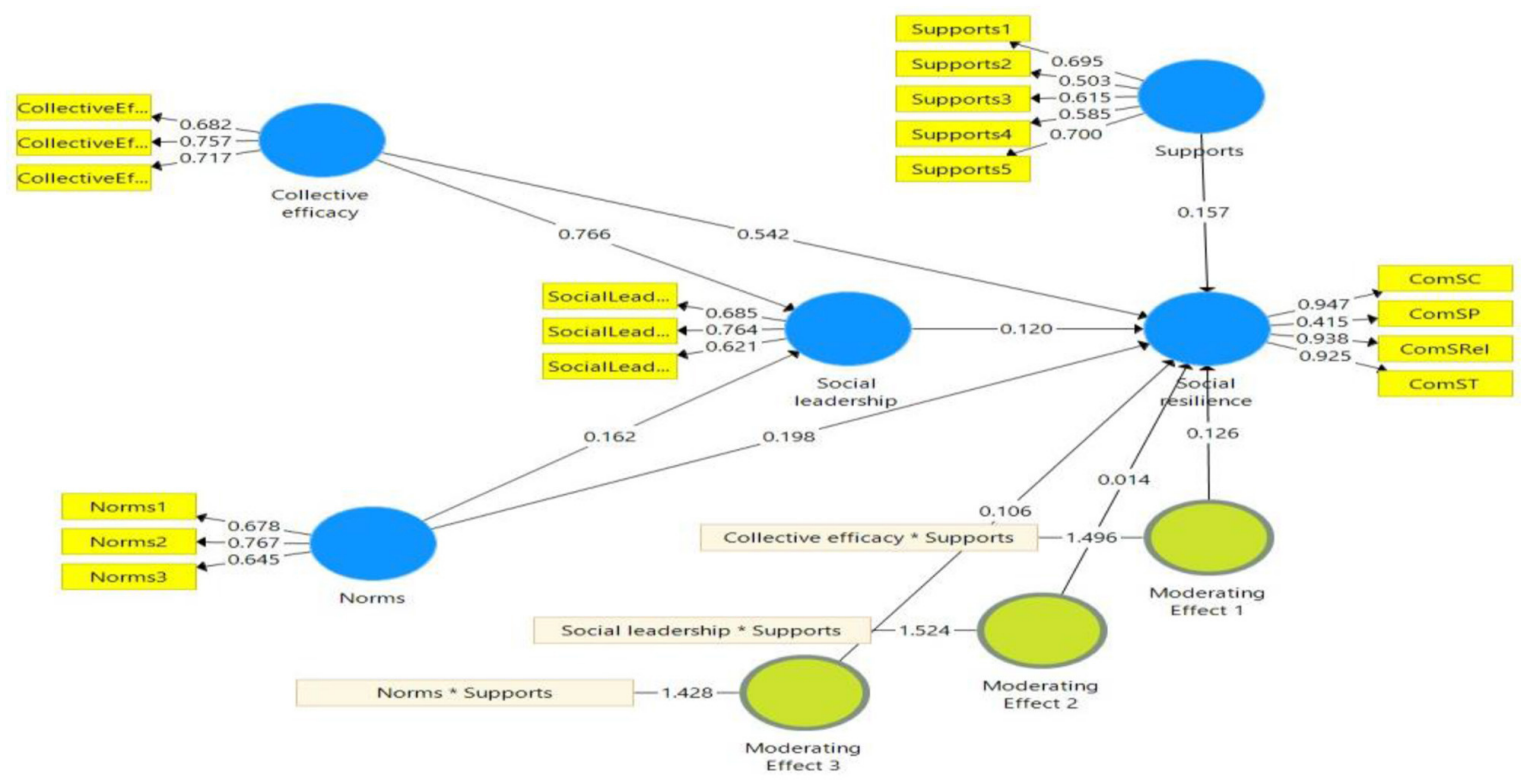
The moderated mediation structural model.

At this stage, the conceptual framework was run using SmartPLS3 to test the hypotheses using a structural model. Because in the conceptual framework of the present study, there was a mediating variable (social leadership) and a moderating variable (governmental supports), structural equation modeling was run to test the hypotheses in two stages. In the first step, a structural model was implemented to estimate the standardized path coefficients. However, at this stage, the significance of the path coefficients is not specified. Therefore, in the second step, the bootstrapping method was employed to estimate the significance of the path coefficients. The bootstrapping method uses the T-statistic to estimate the significance of the paths. Table 4 and Figure 2 demonstrate the summary testing direct and indirect (mediation and moderation) hypotheses.

**Table 4:** Summary of testing hypotheses.

**Hypothesis**	**Path**	**Beta values**	***t*-value**	***P*-value**	**Result of a hypothesis test**
**Direct hypotheses**					
H1	Collective efficacy -> Social resilience	0.542	9.807	0.001	Supported
H2	Collective efficacy -> Social leadership	0.766	14.822	0.001	Supported
H3	Norms -> Social resilience	0.198	3.579	0.001	Supported
H4	Norms -> Social leadership	0.162	2.884	0.004	Supported
H5	Social leadership -> Social resilience	0.120	2.230	0.026	Supported
**Indirect (mediation) hypotheses**					
H6a	Collective efficacy -> Social resilience	0.092	2.159	0.031	Supported
H6b	Norms -> Social resilience	0.020	1.701	0.089	Rejected
**Indirect (moderation) hypotheses**					
H7a	Moderating effect 1 -> Social resilience	0.126	2.162	0.031	Supported
H7b	Moderating effect 2 -> Social resilience	0.014	0.376	0.707	Rejected
H7c	Moderating effect 3 -> Social resilience	0.106	1.642	0.101	Rejected

The results of testing direct hypotheses showed that the effects of collective efficacy on social resilience against the COVID-19 pandemic (β = 0.542; *p* < 0.01) and social leadership (β = 0.766; *p* < 0.01) are positive and significant. These results support H1 and H2. Estimation of the direct effects of norms on social resilience and social leadership revealed that this variable positively and significantly affected social resilience against the COVID-19 pandemic (β = 0.198; *p* < 0.01) and social leadership (β = 0.162; *p* < 0.01), supporting H3 and H4. The final direct hypothesis was related to the effect of social leadership on social resilience against the COVID-19. The results implied that social leadership positively and significantly affected social resilience against the COVID-19 (β = 0.162; *p* < 0.05), supporting H5.

As was mentioned earlier, we hypothesized that social leadership mediates the relationship between collective efficacy and norms with social resilience against the COVID-19 pandemic. The results of this part of the analysis revealed that the indirect (mediation) effect of collective efficacy on social resilience is positive and significant (β = 0. 092; *p* < 0.05). In other words, the present study has sufficient evidence to support H6a. However, the indirect (mediation) effect of norms on social resilience against the COVID-19 was not statistically significant. Therefore, H6b was rejected. We hypothesized that governmental supports moderate the effects of collective efficacy, social leadership, and norms on social resilience against the COVID-19. The results demonstrated that among these three variables, only the effect of collective efficacy on social resilience (which is moderated by governmental supports) was statistically positive and significant. Therefore, H7a was supported by our results. However, the moderated effects of collective efficacy and norms on social resilience against the COVID-19 were not statistically significant. In other words, H7b and H7c were rejected (Table 4). The results of testing moderated mediation structural model demonstrated that the independent variables were able to predict 62 and 42% of the variance changes of SRCS and SLEC, respectively.

## Discussion

This study examined the effects of collective efficiency and norms on the social resilience of rural Iranians against the COVID-19 pandemic. In the process, social leadership in epidemic conditions mediated the relationship between “collective efficiency in disease control” and “norms” and social resilience. Governmental support was also considered as a moderator of the relationship between three independent variables (collective efficiency in disease control, norms about COVID-19, and social leadership in epidemic conditions) with social resilience against the COVID-19 pandemic. H1 and H2 tests showed a positive and significant effect of collective efficiency in disease control on social resilience against the COVID-19 pandemic and social leadership in epidemic conditions. In other words, the higher the perceived collective efficiency in disease control, the higher the social resilience against the COVID-19 and social leadership in epidemic conditions. Studies by researchers such as Elcheroth and Drury, Lin and Chung, and Stevenson et al. (45–47) supported the positive effect of collective efficacy on social resilience. The result of the H2 test (effect of collective efficiency in disease control on social leadership in epidemic conditions) has also been confirmed by Bamberg et al. (38) and Schulte et al. (39). Considering the positive and significant effect of collective efficiency in disease control on social resilience against the COVID-19 and social leadership in epidemic conditions, it can be concluded that by strengthening the perceived collective efficacy of villagers toward the COVID-19 pandemic, their social resilience to the crisis can be increased. Alizadeh and Sharifi (1) state that some crises, such as COVID-19, are crises at the macro level, and resilience against them requires collective actions and social strategies. In other words, it is usually not possible to respond to them by increasing individual efficiency. In such cases, strengthening the perceived collective efficiency in communities or stakeholders can be a good way to strengthen social resilience. In this regard, it is suggested that collective efficiency in disease control be institutionalized in rural communities using awareness and enlightenment programs. This can be done by the executive arms of health care systems in rural areas. Improving perceived collective efficiency in disease control can not only lead to higher social resilience against the COVID-19 in communities but also positively impact social leadership.

The results of the structural model of the study demonstrated that norms about COVID-19 have a positive and significant effect on social resilience and social leadership in epidemic conditions, which support H3 and H4. This result means that as the desired norm increases, so do the social resilience against the COVID-19 and social leadership in epidemic conditions. Many studies have supported the positive effect of norms on coping behaviors and resilience of societies [see (50, 54, 55)]. In addition, the result obtained for H4 is in line with the findings of Bamberg et al. (38) and Kianmehr et al. (69). Considering the positive and significant effect of norms about COVID-19 on social resilience, it can be inferred that by improving the existing norms in the rural community regarding the corona epidemic, their resilience in the face of this crisis can be improved. According to Yu et al. (50), in analyzing the norms of communities in dealing with the shocks, its two main dimensions, namely social and moral dimensions, should be considered. Therefore, it can be said that one of the practical ways of developing social resilience against the COVID-19 is to strengthen social and moral norms. Ideally, norms act as controllers of behavior. For example, many villagers consider it a moral duty to follow health protocols, violating which may endanger the lives of their fellow villagers in the village and elsewhere. In some cases, individuals are very observant of health protocols because they feel that if they do not do so, they will be punished by the community and those around them. Such control by the norms over the behavior of the villagers during the epidemic will ultimately lead to an increase in their social resilience against the COVID-19 pandemic. In this regard, it is suggested to strengthen the moral and social norms in the field of the COVID-19 pandemic in rural communities using three strategies: (1) fostering personal norm/responsibility of individuals toward their peers; (2) awareness of the benefits or consequences of strict adherence to health protocols; and (3) punishing those who violate the norms of society during COVID-19 pandemic. These three strategies can be carried out by the elites and key informants of villages and field staff of health care systems. It should be noted that mass media and social networks can also play a key role in the successful implementation of the first and second strategies.

The results of the structural model also implied that social leadership in epidemic conditions has a positive and significant effect on social resilience against the COVID-19 pandemic. Thus, the H5 was also supported. The greater social leadership during the COVID-19 pandemic, the greater the social resilience of rural communities to shock. This result has been supported by others [see (1, 7, 60)]. Alizadeh and Sharifi (1) state that social leadership increases the influence and cooperation of members of the rural community with each other in the field of the COVID-19 pandemic. This factor facilitates adaptation strategies reduces copying costs and ultimately leads to social resilience to shock. In this regard, it is recommended that the spirit of collective leadership be strengthened in rural communities. Building mutual trust and collective identity are one of the first steps in developing collective leadership during the COVID-19 pandemic. In other words, all members of the rural community and practitioners of health care systems must act in a way that strengthens internal trust and collective identity. Commitment to consider successes and failures as the result of the collective work of all members of society and to strengthen and enhance the quality of human interaction in the process of forming and sustaining social resilience against COVID-19 are key issues. Continuous and effective communication through various communication channels can be the second step to strengthen social leadership during the COVID-19 pandemic. In this regard, it is suggested that members of rural communities try to activate the most effective and accessible communication channels among themselves and increase the quality of communication between them. Electronic communications and social networks can be used effectively for this purpose.

In H6a and H6b, the mediating role of social leadership in epidemic conditions was tested. The results revealed that the indirect (mediated) effect of collective efficiency on social resilience is positive and significant, which supports H6a. This result means that social leadership in epidemic conditions mediates the relationship between these two variables. However, the indirect (mediated) effect of norms on social resilience against the COVID-19 was not significant (H6b was not supported). In other words, social leadership in epidemic conditions cannot be considered as a mediator of the relationship between norms and social resilience. The results obtained from testing these two hypotheses can be useful in theory and practice. Using collective efficacy reinforcement methods (described in the first paragraph of this section) will increase social resilience against the COVID-19 pandemic. In addition, strengthening the collective efficacy by facilitating social leadership will also lead to the social resilience of villagers against the shock. This is a key result that can be used in social and psychological intervention programs during the COVID-19 pandemic.

The results of testing H7a, H7b, and H7c showed that H7a is the only hypothesis supported by the data. In other words, governmental supports only moderate the effect of collective efficacy on social resilience against shock (H7a was supported). However, this variable did not moderate the effects of social leadership and norms in social resilience (H7b and H7c were rejected).

Given that governmental support positively and significantly moderated the effect of collective efficiency on social resilience, it is suggested that the number and variety of governmental support be increased in the rural areas during the COVID-19 pandemic. Studies by other researchers such as Yazdanpanah et al. (12) and Ratnasingam et al. (62) have also pointed to the importance of government support in vulnerable agricultural and rural communities. Since the outbreak of COVID-19, Iran's rural communities, which are mainly weak economically, have faced new problems due to their inability to market and sell their products. The pressures of international sanctions have also made their economic conditions very fragile and vulnerable. In such circumstances, government support is one of the most important strategies to strengthen social resilience against shock.

## Conclusions

This study resulted in four important conclusions that could be used to encourage social resilience against the COVID-19. The first conclusion was that although three variables collective efficacy, social leadership, and norms have significant direct effects on social resilience against the COVID-19, collective efficacy is considered the most important direct predictor. Second, social leadership can mediate the relationship between collective efficacy and social resilience. Third, governmental support can only moderate the effect of collective efficacy on social resilience. In other words, the effects of social leadership and norms on social resilience are not moderated by governmental support. Fourth, social leadership variables are predicted by collective efficacy and norms. The most important original contribution of this research is to present an innovative moderated-mediation model that explains the mechanism of relationships between predictors of social resilience accurately and realistically. Also, as the main take-home messages of the study, two points should be highlighted: First, social leadership can mediate the effects of norms and collective performance on social resilience. Second, governmental support can only moderate the effect of collective efficiency on social resilience.

It should be noted that the present study had several limitations. First, the present study investigated the social resilience of Iranian villagers toward COVID-19. However, the resilience of rural communities against this disease has economic, physiological, institutional, and even psychological dimensions. It is recommended that future researchers consider other dimensions of resilience as well. Second, the independent variables included in the model of this study were able to predict 62% of the variance changes in social resilience against the COVID-19 pandemic. This shows that other variables that are not present in the model can still increase the explanatory power of the model. In this regard, it is suggested that future researchers contribute to the development of the framework by introducing new variables in the model. Third, the present study uses a self-reporting questionnaire to collect data on social resilience against the COVID-19 and the factors affecting it. This may affect the outcome of the research. In this regard, it is recommended that future researchers at least use complementary methods of data collection along with the self-reporting method. Fourth, social resilience against the corona virus certainly requires the collective actions of the villagers. However, this variable and its determinants have not been examined in present study. Future researchers are recommended to focus on this variable and the role its determinants.

## Data Availability Statement

The raw data supporting the conclusions of this article will be made available by the authors, without undue reservation.

## Ethics Statement

Ethical review and approval was not required for the study on human participants in accordance with the local legislation and institutional requirements. The patients/participants provided their written informed consent to participate in this study.

## Author Contributions

NV, EG, MA, and JS contributed to the conception and design of the study and performed the statistical analysis. MA and NV wrote the first draft of the manuscript. EG and NV contributed to data collection. All authors contributed to manuscript revision, read, and approved the submitted version.

## Conflict of Interest

The authors declare that the research was conducted in the absence of any commercial or financial relationships that could be construed as a potential conflict of interest. The reviewer MM declared a shared affiliation with one of the authors JS to the handling editor at time of review.

## Publisher's Note

All claims expressed in this article are solely those of the authors and do not necessarily represent those of their affiliated organizations, or those of the publisher, the editors and the reviewers. Any product that may be evaluated in this article, or claim that may be made by its manufacturer, is not guaranteed or endorsed by the publisher.
